# Retrospective Use of Whole-Genome Sequencing Expands the Multicountry Outbreak Cluster of *Listeria monocytogenes* ST1247

**DOI:** 10.1155/2021/6636138

**Published:** 2021-04-01

**Authors:** Mihkel Mäesaar, Rafael Mamede, Terje Elias, Mati Roasto

**Affiliations:** ^1^Chair of Food Hygiene and Veterinary Public Health, Institute of Veterinary Medicine and Animal Sciences, Estonian University of Life Sciences, Fr. R. Kreutzwaldi 56/3, 51006 Tartu, Estonia; ^2^Instituto de Microbiologia, Instituto de Medicina Molecular, Faculdade de Medicina, Universidade de Lisboa, Avenida Professor Egas Moniz, 1649-028 Lisboa, Portugal

## Abstract

*Listeria monocytogenes* sequence type 1247 clonal complex 8 caused a prolonged multicountry outbreak in five EU countries: Denmark, Estonia, Finland, France, and Sweden. A total of 22 disease cases were identified with onset of symptoms between July 2014 and February 2019. Five patients died due to, or with, the disease. The retrospective analysis of *L. monocytogenes* isolate VLTRLM2013 revealed the presence of an outbreak-related strain (cgMLST type L2-SL8-ST1247-CT4158) in ready-to-eat fish product more than a year prior to the first outbreak-related cases. Reference outbreak strain and VLTRLM2013 strain were compared using core genome and whole-genome multilocus sequence typing analyses. Genomic level differences of the persistent *L. monocytogenes* strains associated with a prolonged multicountry foodborne listeriosis outbreak are described. It was concluded that the persistent nature of the multicountry outbreak-related *L*. *monocytogenes* strain VLTRLM2013 together with stress island, virulence, and antibiotic resistance genes could potentially be the determining factors for the extensive and prolonged outbreak affecting five European Union countries. Our results support the systematic application of whole-genome sequencing in food and public health surveillance and further encourages its wide adoption.

## 1. Introduction

*Listeria monocytogenes* is capable of surviving and reproducing in various environmental conditions [1] and habitats as well as causes serious infection in humans and animals [2]. It is the causative agent of listeriosis and the most severe human zoonosis with the highest hospitalization (97.0%) and case-fatality (15.6%) rates in the European Union (EU) in 2018, with 2,549 confirmed cases and 14 foodborne listeriosis outbreaks [3].

The ubiquitous nature of the pathogen [4] and the presence of growth niches in the food production environments could potentially be the causes of persistent “in-house” *L*. *monocytogenes* strains emerging in the food processing plants [1]. Cross-contamination in food production plants enables *L*. *monocytogenes* to enter the food production chain and ultimately might lead to the contamination of the food products [5]. Ready-to-eat (RTE) food products, especially fish and fish-derived products, have been shown to be important vehicles for *L*. *monocytogenes* transmission leading to human infection [6, 7], as they are often consumed cold-smoked or without any heat treatment [8] such as salted raw fish products [7]. Therefore, consumption of these products could potentially lead to foodborne listeriosis outbreaks [9, 10].

On the 4^th^ of June 2019, the European Centre for Disease Prevention and Control (ECDC) and European Food Safety Authority (EFSA) published a rapid outbreak assessment (ROA) regarding the multicountry outbreak caused by *L. monocytogenes* sequence type (ST) 1247 clonal complex (CC) 8 [10]. As of the 23^rd^ of May 2019, Denmark, Estonia, Finland, France, and Sweden reported altogether 22 confirmed cases of *Listeria monocytogenes* sequence type ST1247 CC8 outbreak from the period of 2014-2019 [10]. Outbreak investigation pointed to Estonian fish processing company A as a single manufacturer of the products associated with the outbreak [10].

A study conducted in 2012-2013 at the retail level in Estonia [11] collected isolates from RTE fish products including those that originated from the fish processing company A. *L*. *monocytogenes* isolates obtained during this study were stored in the Department of Food Hygiene and Veterinary Public Health at the Estonian University of Life Sciences. We hypothesized that the *L*. *monocytogenes* isolate obtained from this culture collection one and a half years prior to the first confirmed outbreak-related case is part of the same prolonged multicountry outbreak cluster.

## 2. Material and Methods

### 2.1. Bacterial Strain

*L*. *monocytogenes* strain VLTRLM2013 was isolated from company A vacuum-packaged sliced salted salmon product on the 29th of January 2013. The sample was originally collected during a study focusing on the vacuum and modified atmosphere packaged RTE meat and fish products of Estonian origin at the retail level in 2012-2013 [11]. VLTRLM2013 was the only strain that originated from company A from this study. The strain was isolated in the Estonian Veterinary and Food Laboratory in accordance with EVS-EN ISO 11290-1:2000/A1:2004 [12]. The isolated strain was stored at -82°C in Protect microorganism preservation system tubes (Technical Service Consultants, UK) in the Department of Food Hygiene and Veterinary Public Health at the Estonian University of Life Sciences.

Genome sequencing and assembly were performed as described by Mäesaar and Roasto [13]. The isolate was recultivated on blood agar (Biolife Italiana, Italy) at 37°C under aerobic growth conditions. Genomic DNA was extracted using an IndiSpin pathogen kit (Indical Biosciences, Germany). DNA concentration and quality were checked using a Qubit 4 fluorometer with the double-stranded DNA broad-range assay kit (Thermo Fischer Scientific, USA). DNA was sheared using an M220 focused ultrasonicator (Covaris, USA) and the library was prepared using a TruSeq DNA PCR-free library preparation kit (Illumina, USA). DNA extraction and sequencing were performed at the Estonian Veterinary and Food Laboratory using the Illumina MiSeq system with MiSeq reagent kit v3 (600 cycles). The quality of the reads was assessed using FastQC v0.11.9 [14], and trimming was done with Trimmomatic v0.39 [15]. Reads were assembled using SPAdes v3.14.1 [16], and postassembly quality filtering was applied as described by Llarena et al. [17] and Mäesaar and Roasto [13].

The raw sequencing reads, genomic sequences, and genome assembly have been deposited into the National Center for Biotechnology Information (NCBI) databases under the accession numbers SRR11789329, JABGCT000000000.1 and GCA_013302955.1, respectively [13]. The genome assembly was also submitted to the *L*. *monocytogenes* multilocus sequence typing MLST database (https://bigsdb.pasteur.fr/listeria/) [18, 19] under the identification number 48183.

Representative *L*. *monocytogenes* ST1247 reference outbreak strain genome used in this study was previously published by ECDC and EFSA along with the *L*. *monocytogenes* rapid outbreak assessment and is available in the European Nucleotide Archive (ENA) under the accession number ERR2223569 [10]. Genome assembly used in this study is available upon request from ECDC.

### 2.2. Genomic Analyses

Reference outbreak and VLTRLM2013 strains were compared using core genome (cg) and whole-genome (wg) MLST analyses. Institut Pasteur *L*. *monocytogenes* MLST database was used to perform cgMLST analysis with a scheme that included 1,748 genes [18]. The chewBBACA software v2.5.4 [20] was used for wg/cgMLST schema creation, allele calling, and cgMLST extraction with default parameter values. Reference outbreak strain reads were used with VLTRLM2013 as the reference genome for Snippy v4.6.0 variant calling with default parameter values [21]. Genome annotation was performed using Prokka v1.14.6 with UniProt *L*. *monocytogenes* proteome (UP000000817) as annotation reference and default parameter values [22, 23]. Allelic differences determined in the wg/cgMLST analysis were translated and then annotated using Basic Local Alignment Search Tool (BLAST) [24–26]. The presence of virulence, metal, and detergent resistance, stress island, and antibiotic resistance genes in the VLTRLM2013 strain was determined using Institut Pasteur *L*. *monocytogenes* database [18]

All the results from Institut Pasteur's analysis regarding cgMLST, virulence, stress island, metal and detergent resistance, and antibiotic resistance genes can be accessed through the BIGSdb-Pasteur website under the isolate identification number 48183 assigned to the VLTRLM2013 strain.

## 3. Results

The study revealed that *L*. *monocytogenes* strain VLTRLM2013 belonged to the same ST1247 CC8 with deduced PCR-serogroup IIa (4) [13, 18] and the same cgMLST cluster (L2-SL8-CC8-CT4158, Institut Pasteur scheme [18], analysis performed by Institut Pasteur) as the reference outbreak strain [10]. The two strains had three allelic differences (*lmo1630* (*trpC*), *lmo2399*, and *lmo2778*) out of 1,744 loci detected in both isolates based on 1,748 cgMLST scheme [18].

The wgMLST scheme created using chewBBACA and based on VLTRLM2013 and reference outbreak strains consisted of 2,783 genes. Comparison of allelic profiles revealed five allelic differences between the two isolates, out of 2,770 loci detected in both isolates. Three out of the five allelic differences were concordant with the differences found with the Institut Pasteur 1,748 cgMLST scheme [18]. The other two differences were in the loci that code for the restriction endonuclease subunit S and UDP-N-acetylmuromoyl-L-alanyl-D-glutamate--2,6-diaminopimelate ligase proteins. Four out of five allelic differences consisted of SNPs that were detected in the *lmo1630*, *lmo2399*, *lmo2778*, and *murE* genes. All the detected SNPs were nonsynonymous and did not lead to premature stop codons (PMSC). The only non-SNP allelic difference was identified in the locus that codes for the restriction endonuclease subunit S protein, with 76% similarity between the alleles of both strains based on BLASTp alignment.

Out of the 93 virulence-associated genes included in the Institut Pasteur virulence gene scheme [18], 57 were present and likely to be functional (complete coding regions) in VLTRLM2013, including the *inlA* and *inlB* genes that encode proteins associated with host cell invasion [27, 28]. *Listeria* pathogenicity island 1 (LIPI-1) was present, but LIPI-2, LIPI-3, and LIPI-4 genes were not detected in the genome of the isolate VLTRLM2013 [29–32].

None of the 12 metal and detergent resistance genes included in the Institut Pasteur's MLST database scheme were found to be present in the *L*. *monocytogenes* strain VLTRLM2013.

Stress island analysis revealed the presence of the *lmo1800* gene which encodes protein LipA associated with virulence of *L*. *monocytogenes* [33]. Additionally, the analysis detected the presence of the five-gene stress survival islet (SSI-1) that contributes to the growth of the pathogen under suboptimal conditions [34].

WGS analysis also revealed the presence of five genes related to antibiotic resistance: *fosX* (fosfomycins) [35], *lmo0919* (lincosamides) [36], *mprF* (cationic antimicrobial peptides) [37], *norB* (quinolones) [38], and *sul* (sulfonamides).

## 4. Discussion

The benefit of using WGS to enhance surveillance of listeriosis is demonstrated by Van Walle et al. [39]. The study revealed that using molecular typing in combination with epidemiological and food exposure investigations may lead to earlier detection of multicountry outbreak clusters associated with the *L. monocytogenes* [39]. WGS has been successfully applied to investigate several multicountry *L*. *monocytogenes* outbreaks in the EU [9, 10, 40, 41].

Many studies have reported recurrent contamination of food products with persistent *L*. *monocytogenes* strains surviving in food processing plants over months to several years [1, 4, 42–44]. In this study, the persistence was defined as long-term survival, e.g., repeated isolation of *L*. *monocytogenes* strain belonging to the ST1247 in food processing environment and repeated cross-contamination of food products over a long period of time. *L*. *monocytogenes* ST1247 CC8 isolates matching the reference genome of the outbreak strain were isolated from July 2014 to February 2019 [10]. Isolates originated from environmental samples and fish products of Estonian processing company A and also from human patients [10]. *L*. *monocytogenes* ST1247 CC8 strain VLTRLM2013 was isolated in January 2013 from the same company's vacuum-packaged sliced salted salmon product. Previous studies conducted in Estonia have confirmed that RTE fish products are high-risk category products regarding *L*. *monocytogenes* [7, 11]. Retrospective analysis revealed the presence of an outbreak-related *L*. *monocytogenes* strain more than a year prior to the first outbreak-related cases. The strain VLTRLM2013 was assigned to the same cgMLST cluster (L2-SL8-CC8-CT4158) as the Danish representative outbreak strain isolated in 2017. The two strains had only three cgMLST allelic differences using Institut Pasteur scheme [18], which is similar to the core genome evolutionary rate previously reported by Moura et al. [18]. cgMLST analysis confirmed the hypothesis that *L*. *monocytogenes* strain VLTRLM2013 falls within the outbreak cluster detected by the ECDC and EFSA [10]. Additionally, the wgMLST schema created based on both isolates enabled to extend the results obtained with the cgMLST schema from Institut Pasteur. The wgMLST analysis revealed only five allelic differences between the two isolates. The number of allelic differences indicates that the two isolates are very similar and further supports the set hypothesis.

*L*. *monocytogenes* ST1247 CC8 outbreak-related isolates have been circulating for over six years, from 2013 to 2019, causing a prolonged multicountry outbreak of 22 listeriosis cases [10]. In the period of 2014-2018, altogether, nine *L*. *monocytogenes* ST1247 strains were detected in Estonia by the Health Board [45]. The presence of SSI-1, which has been previously associated with persistent *L. monocytogenes* strains [43], could be the potential cause of the prolonged outbreak associated with the *L*. *monocytogenes* ST1247 CC8 isolate VLTRLM2013. The presence of SSI-1 could give a competitive advantage to the isolates in growth at low pH and high salt concentrations, therefore making the isolate more lasting in suboptimal conditions, for example, in food environments [34].

From all the confirmed listeriosis cases (*n* = 22) caused by *L*. *monocytogenes* ST1247, five patients have died due to, or with, the disease [10]. Virulence of strain VLTRLM2013 could be potentially related to the presence of 57 known virulence genes revealed by the virulence factor analysis. Findings included *inlA* and *inlB* genes, related with host cell invasion [27, 28]. Schubert et al. [27] demonstrated that listerial protein InlA has a role in bacterial adhesion and epithelial cell invasion in the human intestine. Latter is complemented by the ability of InlB to promote invasion of cells not permissive for InlA [28]. The isolate VLTRLM2013 harbors LIPI-1 genes, but lacked LIPI-2, LIPI-3, and LIPI-4 genes and also did not possess metal and detergent resistance genes. Additionally, the strain VLTRLM2013 had the *lmo1800* gene which encodes protein LipA associated with the virulence of *L*. *monocytogenes* in vivo [33]. The genome of the strain VLTRLM2013 had antibiotic resistance genes conferring resistance to fosfomycins, lincosamides, cationic antimicrobial peptides, quinolones, and sulfonamides.

The persistent nature of the multicountry outbreak-related *L*. *monocytogenes* strain VLTRLM2013 together with stress island, virulence, and antibiotic resistance genes could potentially be the reasons of such an extensive prolonged outbreak affecting five EU countries [10].

## 5. Conclusions

Retrospective use of whole-genome sequencing analyses enabled to expand the knowledge of the *L. monocytogenes* ST1247 CC8 multicountry outbreak cluster. In the present study, we offered insight into the genomic level differences of the persistent *L. monocytogenes* strains associated with the prolonged multicountry foodborne listeriosis outbreak. Involving different stakeholders such as state food control and public health institutions, universities, and other research facilities ensures the prompt epidemiological analysis of the foodborne outbreaks.

Systematic and timely application of WGS in routine surveillance contributes to the effectiveness of foodborne outbreak investigations, therefore, helping to prevent and/or diminish foodborne outbreak-related disease cases. Regarding the latter, the importance of routine WGS analysis and genomic data sharing should not be underestimated.

## Data Availability

The authors confirm that all the supporting data, code, and protocols have been provided within the article.
